# The use of birds as pets in Mexico

**DOI:** 10.1186/s13002-017-0161-z

**Published:** 2017-06-13

**Authors:** Blanca Roldán-Clarà, Víctor M. Toledo, Ileana Espejel

**Affiliations:** 1 Laboratorio de Etnoecología, Instituto de Investigaciones en Ecosistemas y Sustentabilidad (IIES) Universidad Nacional Autónoma de México, Morelia, Michoacán Mexico; 2 Universidad Autónoma de Baja California (UABC), Carretera Transpeninsular Ensenada - Tijuana No. 3917, Colonia Playitas, C.P. 22860 Ensenada, Baja California Mexico

**Keywords:** Bird-keepers, Ethno-ornithology, Mexican birds, Traditional bird use

## Abstract

**Background:**

The use of birds as pets has been a historical tradition in Mexico since prehispanic times. It has survived through bird traders, called *pajareros*, which is a local name given to the trade (derived from *pájaro*, the Spanish word for bird). However, the trade of birds has not been sufficiently described; therefore, the goal of this paper is to analyze the bird trade in Mexico using the components of an ethnoecology scheme known as *kosmos-corpus-praxis* complex.

**Methods:**

Qualitative research techniques were used, including ethnographic immersion, participative research, and interviews of 79 *pajareros* in 22 localities in nine Mexican states.

**Results:**

The activity of the *pajareros* occurs within their households, with each member having distinct roles. The roles involved in bird trading are capturing, acclimation, maintenance, and sale. Their assignment depends on gender, age, and residential location (rural or urban). Beyond their households, the *pajareros* are well organized in trade unions and are represented by a leader, who acts as an intermediate between them and the government officers who are involved in the authorization of federal permits. The *pajareros* use 96 species of birds, most of which are native to Mexico. Practicing the trade requires highly specific knowledge of the biology, ecology, habitat, nutrition, diseases, and behavior of the birds, as well as the abiotic components of their environment, such as climatology and geography. The cosmovision of *pajarero* households is embedded in their identity, making them proud of their trade.

**Conclusions:**

Our paper provides the first comprehensive description of the *pajarero* trade, showing evidence of local communitarian management in the places where the wild birds are captured.

## Background

The use of wild fauna is a tradition in many communities across several countries, including their use for household consumption (e.g., [1–4]). The use of wild birds is one of many examples of autochthonous use [5], and Mexico is one of the countries in which the use of wild fauna in general (and the use of wild birds in particular) is a culturally and economically important subsistence activity [6]. Ecologists and non-governmental organizations (NGOs) have demonized the capture and confinement of wild birds; this reaction is, in part, due to misinformation regarding the ethnographic origins of the activity. Such a lack of information about the wild bird trade reflects a lack of research providing an in-depth description of it. Therefore, our paper is the first one to describe the bird trade in Mexico; a trade that, despite the negative attitude of NGOs, has subsisted with the support, regulation, and management of the Mexican government. Our research focused on birds that are kept in confinement as pets [7–9] because of their beauty and lively coloration, melodious songs, ability to imitate words, attachment to humans, or all of these reasons [10, 11].

At present, the most commonly used birds are the passerines (order Passeriformes), and the bird market is mostly domestic [12]. Most wild birds are captured in their natural habitat and a small fraction of them are bred in captivity for their commercialization [13]. In Mexico, the bird traders are called *pajareros* (derived from *pájaro*, the Spanish word for bird), and they have maintained the cultural tradition of capturing and maintaining wild birds for sale; this is an activity that, according to historical documents, is of Prehispanic origin [14, 15]. Among the few accounts of the *pajarero* activity in Mexico, the best reports are by Mellink and collaborators [16–18], who described the use of wild birds in the Potosino-Zacatecano Plateau region in north central Mexico. Research on this topic in Latin America is very scarce, and most of the studies consist of birds on the useful bird species list. There are few studies with qualitative data describing the social characteristics of bird users or depicting the interesting capture techniques that they have developed. These studies do not include literature on the extraordinary traditional bird knowledge that these people have. More research regarding this activity has been published for Brazil [19], despite the fact that birds are used as pets in all Latin American countries (e.g., [20]).

The scarcity of information on the human processes involved in the traditional activity of *pajareros* in Mexico urges a complete description of the trade using an ethnoecological scheme [21]. Ethnoecology is the study of the indivisible triad made up of the system of beliefs (the *kosmos*), accumulated knowledge (the *corpus*), and productive practices (the *praxis*) of social groups. Ethnoecological analysis makes it possible to comprehensively understand the established relationships between social groups, the processes of interpretation and representation, and the use or management of natural resources and their appropriation [22, 23]. Consequently, in order to provide a complete view of the complexity of the interactions among cultures, natural resource appropriation, and nature itself, the ethnoecological approach encompassing cosmovision, knowledge, and the utilization or management of natural resources must be interdisciplinary [24] and it must have a comprehensive holistic vision [22, 23, 25, 26].

Local bird capturers and traders have rarely been studied from a scientific, neutral point of view in Latin America. Most of the reports are provided by the organizations that are interested in impeding bird capture [27]. NGOs have no interest in how the bird trade affects the ancestral traditions of local communities. We could not found any reports describing the ethnographic issues of this activity in detail. Therefore, the goal of our present work is to analyze the bird trade in Mexico using the components of an ethnoecology scheme.

## Method

To understand the motivations and beliefs involved in the activities of the Mexican *pajareros*, we used qualitative research techniques. Qualitative techniques render descriptive data, such as the words used by people, their observable behaviors, and what they perceive as significant [28, 29]. For our qualitative research to be possible, worthy of attention, and rigorous, we followed the recommendation of Baxter and Eyles [30]: “purposeful sampling, prolonged engagement, persistent observation, triangulation, thick description, and mechanically recorded data.”

Three fieldtrips were made during 2013. The first trip was made to establish contact with a key player, a *pajareros* leader; the second trip occurred during May to August, and the third one occurred in December. A later 4-week fieldtrip was made during the summer of 2016; this trip was mainly used for interviewing women in *pajarero* households and observing the practices involved in the capture, acclimation, maintenance, and nursing of birds. Ethnographic immersion [31] –i.e., spending time in the *pajarero* households and conducting direct participatory observations [21, 32]– occurred in the *pajarero* homes from 22 localities in nine states of Mexico (Table 1); these visits were made during wild bird capture in the cloud forests or grasslands, during bird sale in the fixed markets, streets, and ambulatory markets (*tianguis*), during sporting events or religious ceremonies, and in the meetings of the associations (unions) of *pajareros*. Participatory observation involves social interaction with the informants in their contexts and the acquisition of data in a systematic and nonintrusive way [29]. Throughout the ethnographic immersion, a field logbook [33], photographs and videotapes were compiled. The main result of spending time with the informants was the generation of reciprocal rapport.

**Table 1:** Localities visited during fieldtrips and interviews by state

State	Number interviews	Locality
Ciudad de Mexico	0	Chalco
		México City
State of Mexico	29	Cuautitlán
		Ecatepec
		Jilotepec
		La ConchaC
		San Bartolo Morelos
		Tenancingo
Guanajuato	1	Celaya
Hidalgo	4	Tulancingo
Jalisco	6	Guadalajara
		Mexticacán
		Tepatitlán
Puebla	18	Amixtlan
		Chipaguatlán
		Puebla capital
		Tlacotepec
Querétaro	4	San Juan del Rio
		Ciudad de Querétaro
San Luis Potosí	2	Enramadas
		Santa María del Río
Veracruz	15	Capulines
		Roca de Oro
TOTAL	79	

Semi-structured interviews [28, 32, 34] were applied as a qualitative technique, as they allow for queries on and an understanding of the motivations and beliefs behind the actions of the *pajareros*. Sampling was mainly conducted by the “snowball” technique through three key informants who were leaders of three *pajarero* unions; these informants subsequently introduced the first author to the other interviewees [21]. Interviews were suspended when a saturation of information was reached [32]. All information was obtained with the participants’ informed consent [35]. All interviews were tape-recorded and were transcribed with a word processor.

All bird species were identified by the first author by direct observation of the birds in cages using her experience and proper bird guides [36–38]. The scientific names were updated using the Birds of North and Middle America Checklist (7th edition), the official source on the taxonomy of birds. This list is produced by the committee of the American Ornithological Society: North American Classification Committee (NACC), which was formed by the merger of the American Ornithologists’ Union and the Cooper Ornithological Society.

The software Atlas.ti (version 5.0) was used for analyzing the contents of the interviews, and the field logbooks were manually analyzed. The analysis consisted of a stepwise formulation of the categories from an exhaustive review of the interview texts [39]. The analysis was descriptive for identifying and cataloguing reality by means of the definition of categories or classes of its elements. The categories were chosen through thematic units [40].

## Results and discussion

### The trade roles of *pajareros*

**Fig. 1:**
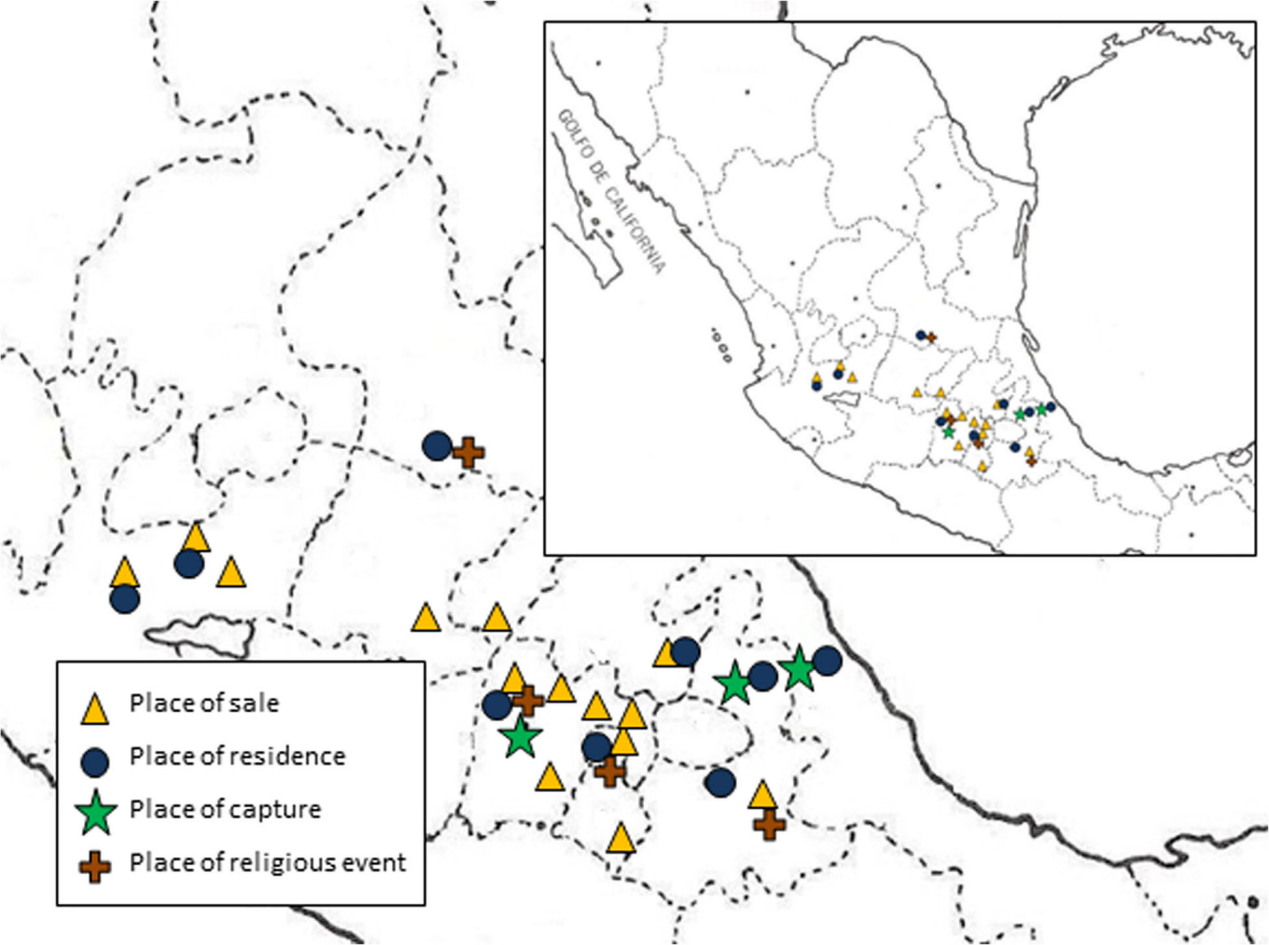
Localization of places visited in central Mexico

The trade of *pajareros* is complex, and it is carried out by specialists playing a particular, but dynamic, role that is assigned according to gender and age. All activities are made within the *pajarero* households –domestic units– in which all members play a role in the bird trading activities. Different members of the *pajarero* households are in charge of capturing or acclimating the birds, manufacturing the cages and other tools, acquiring and retailing the birds, and maintaining the birds (Fig. 1). In Bolivia and Peru, the parrot trade was also found to be family–related [20, 41]. Role assignment by age and gender has been observed in other subsistence activities [42], and in the case of the bird trade studied by Retana Guiascón and collaborators [43], women and children were in charge of capturing and feeding the fledglings.

### Organization

**Fig. 2:**
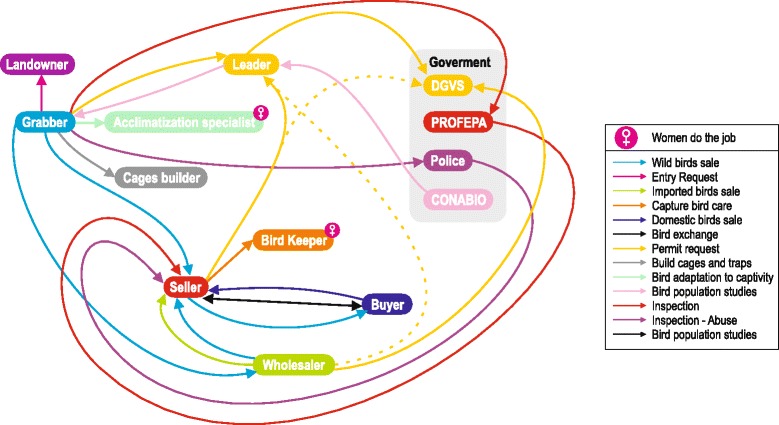
Diagram showing the interactions among the main players involved in bird trade-related activities. DGVS: *Dirección General de Vida Silvestre* (General Direction of Wildlife), PROFEPA: *Procuraduría Federal de Protección al Ambiente* (Federal Attorney General for Environment Protection). CONABIO: *Comisión Nacional para el Conocimiento y Uso de la Biodiversidad* (National Commission for Knowledge and Use of Biodiversity). Own elaboration, Design by Ixchel True

*Pajareros* are well-organized in several trade unions, in which the presidents act as representatives of the union members in government and other instances. All union members interact internally with each other and, through the union, they interact with landlords, buyers, and governmental officers (Fig. 2).

**Fig. 3:**
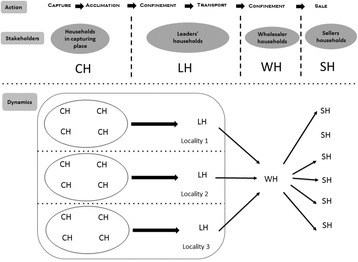
Diagram showing the actions, stakeholders, and dynamics involved in the households of the *pajarero* trade

The *pajarero* trade is a well-established production chain, beginning with the capture of wild birds, continuing with their acclimation and maintenance, and finishing with their sale (Fig. 3). At the community scale or micro-region, the households and leaders in charge of accumulating the captured birds are linked to the production chain; they are sometimes also involved in the acclimation and maintenance of the birds and, if necessary, in transporting the birds to the localities of wholesaler households, which act at a regional scale.

### *Pajarero p*rofile

**Fig. 4:**
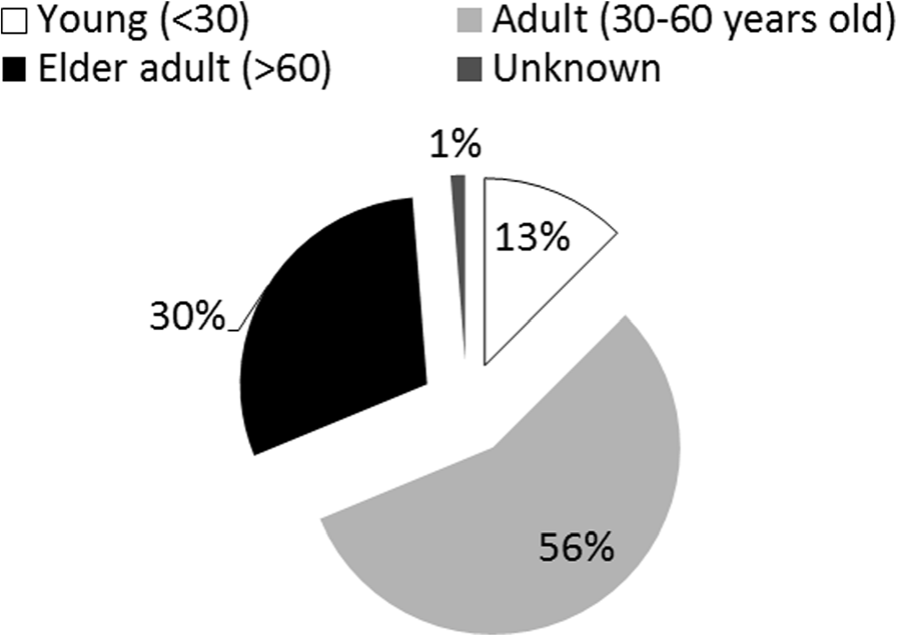
Percentage of interviewees by age category

**Fig. 5:**
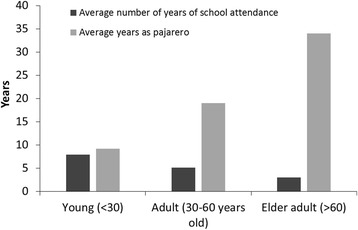
Average years of school attendance and years of experience in the trade for the interviewees by age category

**Fig. 6:**
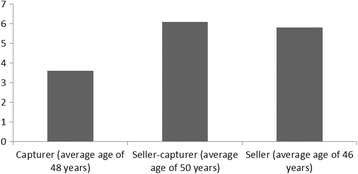
Years of school attendance by activity in the *pajarero* trade

According to official data from the General Direction of Wildlife (DGVS by its Spanish acronym) of the Ministry of the Environment and Natural Resources (SEMARNAT by its Spanish acronym), there were 568 *pajareros* holding permits for capturing wild birds in 2016. Of the 79 *pajareros* interviewed in eight states of Mexico, most [50] were men and 29 were women. Of these same *pajareros*, 18 were capturers, 21 were sellers, 23 performed both activities, and 17 were women in charge of acclimating and keeping the birds. Most of the interviewed *pajareros* were between 30 and 60 years old (Fig. 4). The young informants (under 30 years of age) had a higher educational level than the adults or elders; only one informant had never attended school, and all the young men had completed middle school (two of them had one or more years of high school education). Of the interviewed adults (30–60 years of age), 42% had an incomplete elementary school education, 16% had completed elementary school, and 27% had completed middle school. Of the interviewed elder adults (over 60 years of age), 67% did not complete elementary school and none had completed middle school (Fig. 5). Compared to the average educational level in Mexico, where according to the Organization for Economic Co-operation and Development (OECD), 63% of the population between 25 and 64 years of age has completed a middle school education [44] and 16% of the mature adult population has completed a middle school education [45], *pajareros* have a lower than average educational level, and capturers –who mostly live in rural zones– have a lower educational level than sellers –who are mostly inhabitants of urban zones (Fig. 6). The latter figures agree with the fact that the rural population in Mexico has a lower educational level than the urban population [46]. This observation is similar to other countries; for instance, in Piaui, Brazil, Silva Souto and collaborators [47] also found that bird trappers had low schooling. The mature *pajareros* have more experience in the trade than the young or adult *pajareros* (Fig. 5). The *pajareros* who are capturers and sellers, or only sellers, have been in the trade longer than the *pajareros* who are only capturers. It was deduced that the trade is made up of roles along a *continuum* of activity; there is no sharp line between capturers and sellers, which is similar to what was found for parrot users in Peru and Bolivia, who oftentimes have dual roles [20]. Older sellers were capturers earlier in their lives, when they were young and had the energy to hike in the field; hence, these sellers have had longer careers as *pajareros* than the others.

**Fig. 7:**
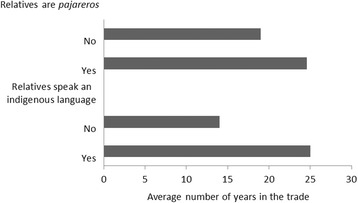
Years in the trade by ethnic origin of the household

**Fig. 8:**
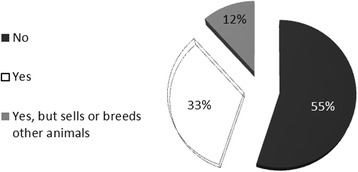
Percentage of *pajareros* exclusively dedicated to the trade

**Fig. 9:**
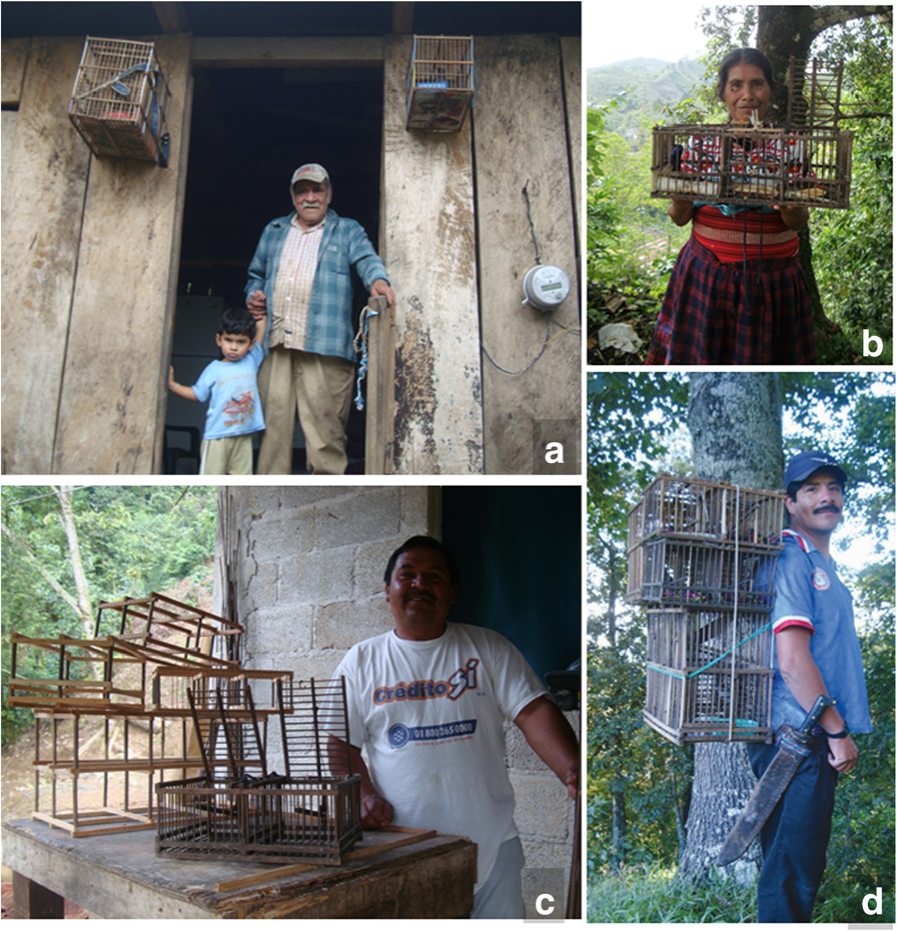
Photographs of *pajareros*. From left to right and from top to bottom are of (**a**) capturer in front of his house in the mountains of Veracruz, (**b**) capturer in the mountains of, Puebla (**c**) manufacturer of cages and traps in the Sierra de Puebla, and (**d**) capturer of slate-colored solitaires (*Myadestes unicolor*) with his trap in the Sierra de Veracruz. Photographs: Blanca Roldán-Clarà

**Fig. 10:**
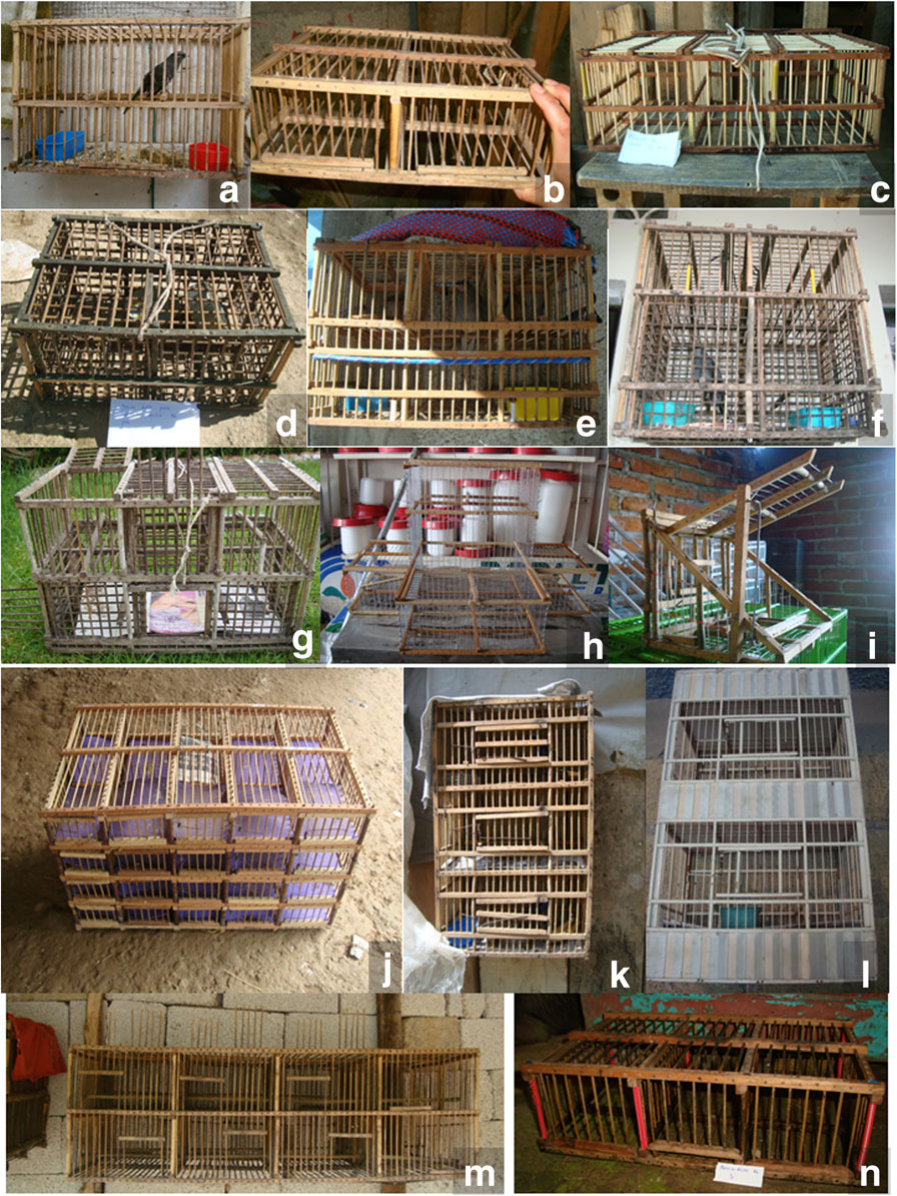
Sample of frameworks used by the *pajareros*. **a** home trap (*Jaula casera*), **b** field trap (*Jaula montera*), **c** trap without a song bird lure (*Pagua* or *Sorda*), **d** double cage for a song bird lure (*Jaula doble* or *Jauloncillo para cabresto*), **e** trap with a song bird lure compartment (*Trampa con cabresto*), **f** double trap with a song bird lure (*Trampa asordada doble*), **g** double trap with a song bird lure compartment (*Trampa de caballete doble*), **h** Yucatec trap (*Trampa yucateca*), **i** Triangular trap (*Trampa triangular*), **j** Transporting cage (*Transportadora*), **k** three-tier cage (*Jaula para tercio*), **l** cage with an elevator (*Jaula garrotera*), **m** large cage (*Jaulón*), **n** cage for recently captured birds (*Recibidora*)

**Fig. 11:**
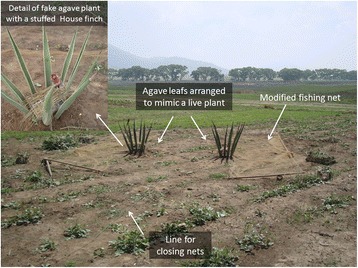
Photograph illustrating an example of the stage mounted by *pajareros* for capturing house finches (*Haemorhous mexicanus*). Photographs: Blanca Roldán-Clarà

**Fig. 12:**
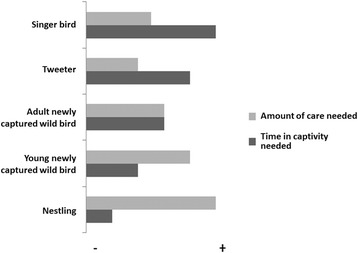
Diagram showing the amount of care and time needed in captivity by the type of acclimated wild bird

**Fig. 13:**
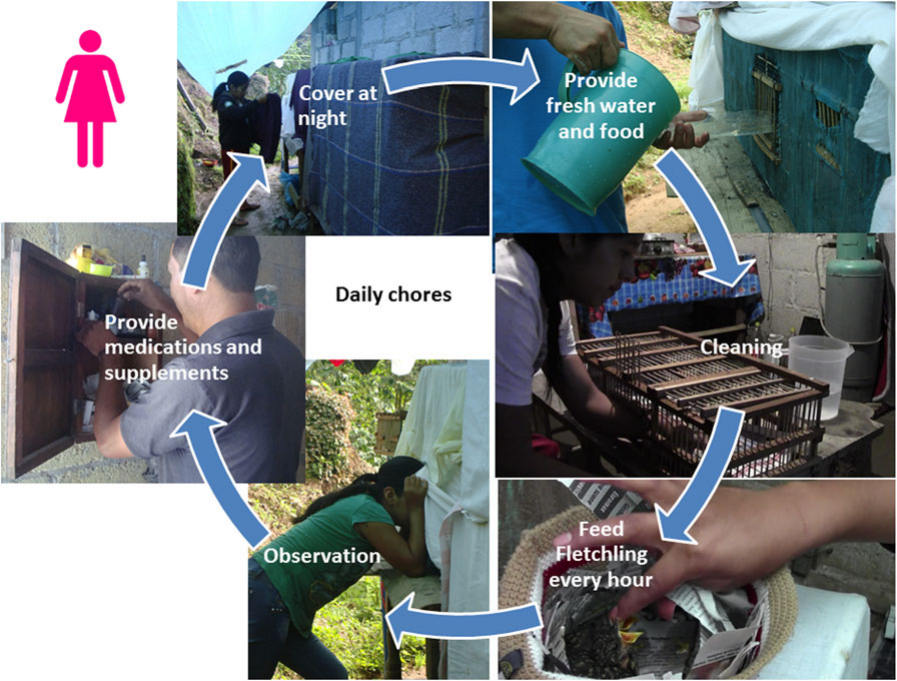
Daily chores needed for maintaining and acclimating birds in captivity

**Fig. 14:**
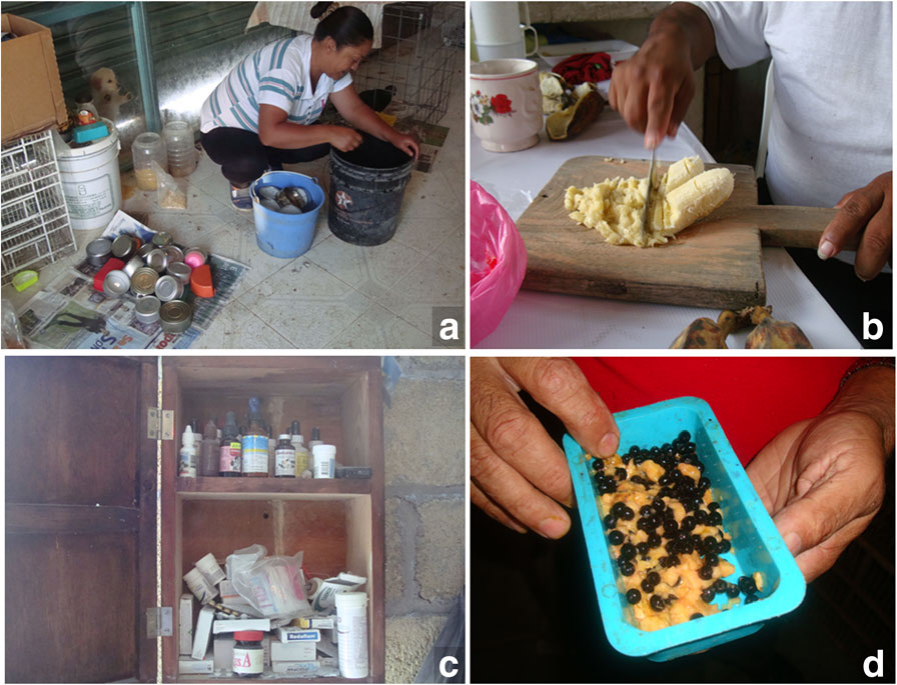
Photographs of *pajareros*. From left to right and from top to bottom: (**a**) bird keeper woman cleaning feeders, (**b**) preparation of plantain for feeding birds, (**c**) medicine cabinet, and (**d**) bird food prepared with plantain and berries. Photographs: Blanca Roldán-Clarà

**Fig. 15:**
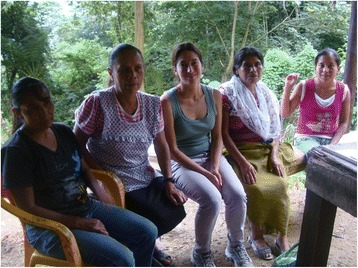
Photograph showing the first author with four specialists in bird acclimation in Amixtlán, Puebla

**Fig. 16:**
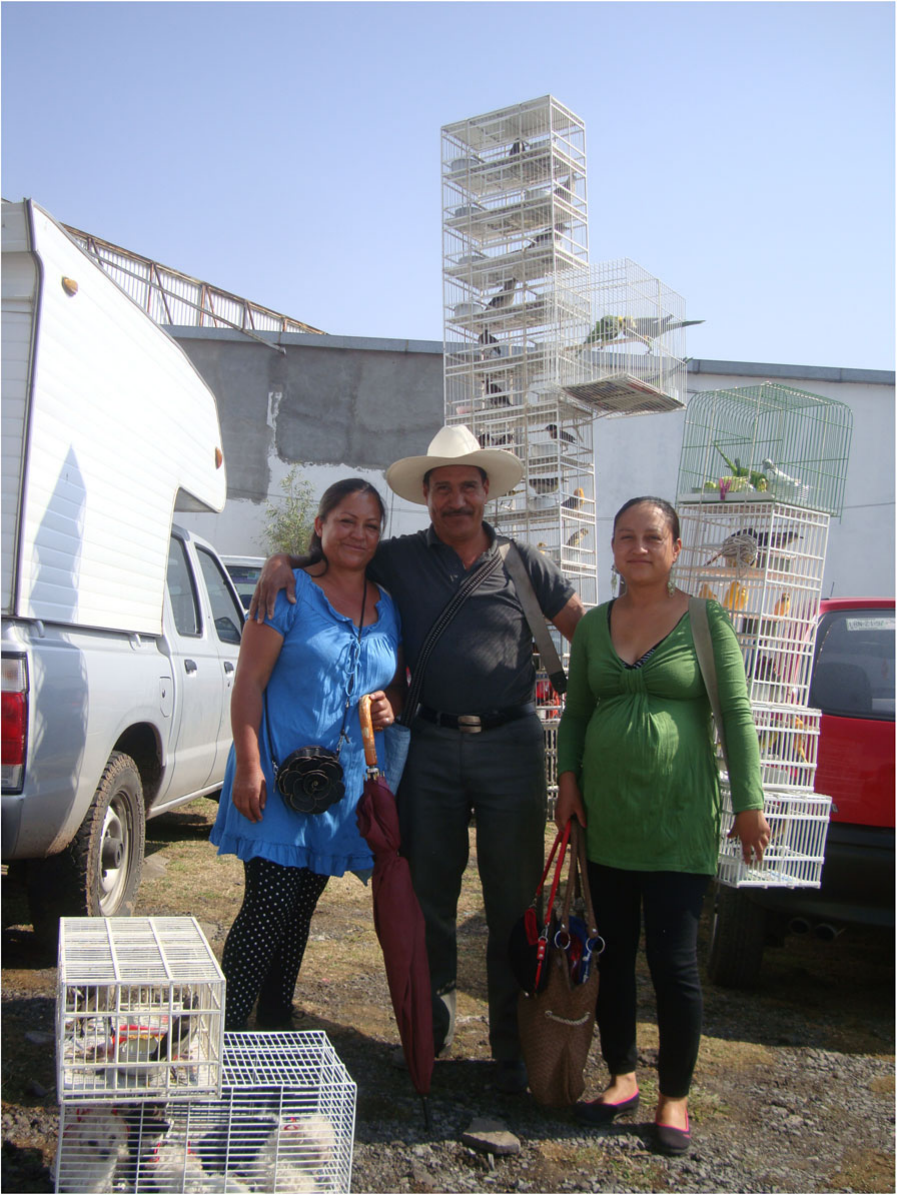
Family of bird sellers from San Bartolo Morelos, State of Mexico

**Fig. 17:**
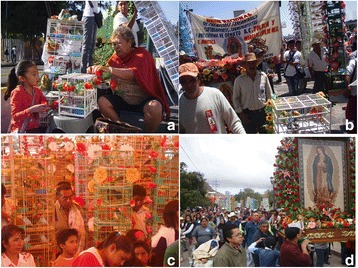
Different moments in the three pilgrimages we studied. **a** Woman and girl decorating a cage with plastic flowers. **b** Pilgrimage to the Basilica of Guadalupe. **c** Pilgrimage to San Bartolo Morelos, State of México; **d** Pilgrimage to Santa María del Río, San Luis Potosí. Photographs: Blanca Roldán-Clarà

The *pajarero* trade is predominantly family-related, given that 80% of the *pajareros* had at least one near relative that is either active or was formerly active in the trade. Having a relative in the trade is associated with having a longer career as a *pajarero* (Fig. 7). Over half of the interviewees (58%) belonged to a native ethnic group –Totonac or Otomi– because they can either speak a native language or have a close relative that speaks a native language. The *pajareros* with native ethnic origins have more experience in the trade (Fig. 7).

The *pajarero* trade is often supplementary, as was found in the Bolivian and Peruvian parrot trade, where the *pajareros* were also tour guides, carpenters or students [20]. Most interviewees have alternative sources of income, particularly among capturers (Fig. 8). The most plausible explanation for this fact is that capture cannot be made in all seasons, while birds can be sold throughout the year. The alternative income of capturers comes from farming activities (such as coffee harvesting) or, for sellers, from selling other commodities, such as prepared food. Therefore, despite the fact that *pajareros* may live in urban centers, they follow a strategy of multiple jobs and the integration of different productive activities, as is common in the native and rural communities of Mexico [23].

### Praxis

#### Bird species managed by *pajarero* households

In Mexico, there are 1123 or 1150 species of wild birds [48]. *Pajarero* households predominantly manage two classes of birds: wild birds captured in their original natural habitats and domestic birds bred in captivity. We identified 96 species of birds from 24 families that are managed by *pajareros*, of which 85 are native to Mexico (7.4% of the country’s total) and only two species –the Yellow-headed Parrot and the Painted Bunting– are categorized as “Endangered” or “Near Threatened” in the Red List of the International Union for Conservation of Nature [49]. Similarly, in Peru, most of the birds were common species and were not threatened [41]. The most represented families were Cardinalidae (43% of the species from this family exist existing in Mexico) and Icteridae (36% of the total in Mexico), each with 13 species, followed by Turdidae (21%), Emberizidae (11%), Fringillidae (44%), Psittacidae (36%), Corvidea (29%) and Mimidae (33%) (Table 2). All these bird families belong to the passerines, with the exception of Psittacidae [50]. The bird families and the proportion of birds are similar to those reported in other studies in Mexico and Latin America [51–56]. For example, passerine families are the most common bird families in Mexico City, and the most common bird families in the Estate of Mexico markets were Cardinalidae, with five species, followed by Turdidae, Icteridae, Fringilidae, with four species each [57]. In the review from Alves and collaborators [53] in Brazil, the bird family with the largest number of traded species was Emberizidae, followed by Psittacidae, Thraupidae, and Icteridae. In a recent study in Floriano, Piauı’ State, Brazil, the most common families found in markets were Thraupidae, Icteridae, Fringillidae, and Turdidae [47]. In Costa Rica, the main families were Psittacids, Emberizidae, Ramphastidae, Turdidae, Fringillidae, Thraupidae, Anatidae, Strigidae and Icteridae, in that order [58] and in Venezuela, they were Emberizidae, Psittacidae and Icteridae [59]. We have found a difference in Peru, as the available articles [41, 60] show a high number of Psittacid specimens on a national level, whereas in Mexico, the majority of the reported birds are passerines.

**Table 2:** Species of birds used by *pajareros* in Mexico

Family	Common name	Scientific name^a^
Phasianidae	Domestic Chicken	Gallus gallus domesticus*
Accipitridae	Common Black-Hawk	*Buteogallus anthracinus*
Columbidae	Rock Pigeon	*Columba livia domestica*
	African Collared-Dove	Streptopelia roseogrisea*
	White-winged Dove	*Zenaida asiatica*
	Mourning Dove	*Zenaida macroura*
Trochilidae	Hummingbird	*Trochilidae*
Momotidae	Motmot	*Momotidae*
Ramphastidae	Emerald Toucanet	*Aulacorhynchus prasinus*
	Keel-billed Toucan	*Ramphastos sulfuratus*
Picidae	Acorn Woodpecker	*Melanerpes formicivorus*
	Gray-breasted Woodpecker	*Melanerpes hypopolius*
Falconidae	American Kestrel	*Falco sparverius*
Psittacidae	Olive-throated Parakeet	*Aratinga nana*
	Orange-fronted Parakeet	*Aratinga canicularis*
	White-crowned Parrot	*Pionus senilis*
	Red-lored Parrot	*Amazona autumnalis*
	Yellow-headed Parrot	*Amazona oratrix*
	Monk Parakeet	Myiopsitta monachus*
	Lovebird	Agapornis sp.*
	Budgerigar	Melopsittacus undulatus*
Cacatuidae	Cockatiel	Nymphicus hollandicus*
Tyrannidae	Great Kiskadee	*Pitangus sulphuratus*
Corvidae	Black-throated Magpie-Jay	*Calocitta colliei*
	White-throated Magpie-Jay	*Calocitta formosa*
	Green Jay	*Cyanocorax yncas*
	Purplish Backed Jay	*Cyanocorax beecheii*
	Western Scrub-Jay	*Aphelocoma californica*
	Steller’s Jay	*Cyanocitta stelleri*
	Common Raven	*corvus corax*
Turdidae	Eastern Bluebird	*Sialia sialis*
	Brown-backed Solitaire	*Myadestes occidentalis*
	Slate-colored Solitaire	*Myadestes unicolor*
	Black-headed Nightingale-Thrush	*Catharus mexicanus*
	Spotted Nightingale-Thrush	*Catharus dryas*
	Black Thrush	*Turdus infuscatus*
	Clay-colored Thrush	*Turdus grayi*
	American Robin	*Turdus migratorius*
	Rufous-backed Robin	*Turdus rufopalliatus*
Mimidae	Northern Mockingbird	*Mimus polyglottos*
	Tropical Mockingbird	*Mimus gilvus*
	Long-billed Thrasher	*Toxostoma longirostre*
	Curve-billed Thrasher	*Toxostoma curvirostre*
	Crissal Thrasher	*Toxostoma crissale*
	Blue Mockingbird	*Melanotis caerulescens*
Sturnidae	European Starling	Sturnus vulgaris*
Bombycillidae	Cedar Waxwing	*Bombycilla cedrorum*
Parulidae	Gray-crowned Yellowthroat	*Geothlypis poliocephala*
Ptilogonatidae	Gray Silky-flycatcher	*Ptilogonys cinereus*
	Phainopepla	*Phainopepla nitens*
Thraupidae	Blue-gray Tanager	*Thraupis episcopus*
Emberizidae	Blue-black Grassquit	*Volatinia jacarina*
	White-collared Seedeater	*Sporophila torqueola*
	Red-legged Honeycreeper	*Cyanerpes cyaneus*
	Yellow-faced Grassquit	*Tiaris olivaceus*
	Chestnut-capped Brush-Finch	*Arremon* sp
	Sparrow	*Spizella* sp
	White-crowned Sparrow	*Zonotrichia leucophriys*
	Yellow-eyed Junco	*Junco phaeonotus*
Cardinalidae	Northern Cardinal	*Cardinalis cardinalis*
	Pyrrhuloxia	*Cardinalis sinuatus*
	Yellow Grosbeak	*Pheucticus chrysopeplus*
	Rose-breasted Grosbeak	*Pheucticus ludovicianus*
	Black-headed Grosbeak	*Pheucticus melanocephalus*
	Blue Seedeater	*Amaurospiza concolor*
	Evening Grosbeak	*Coccothraustes vespertinus*
	Blue Grosbeak	*Passerina caerulea*
	Lazuli Bunting	*Passerina amoena*
	Indigo Bunting	*Passerina cyanea*
	Varied Bunting	*Passerina versicolor*
	Painted Bunting	*Passerina ciris*
	Orange-breasted Bunting	*Passerina leclancherii*
Estrildidae	Munia	Lonchura striata domestica*
	Zebra Finch	Taeniopygia guttata*
Icteridae	Red-winged Blackbird	*Agelaius phoeniceus*
	Yellow-headed Blackbird	*Xanthocephalus xanthocephalus*
	Great-tailed Grackle	*Quiscalus mexicanus*
	Bronzed Cowbird	*Molothrus aeneus*
	Brown-headed Cowbird	*Molothrus ater*
	Shiny Cowbird	*Molothrus bonariensis*
	Yellow-tailed Oriole	*Icterus mesomelas*
	Altamira Oriole	*Icterus gularis*
	Scott’s Oriole	*Icterus parisorum*
	Baltimore Oriole	*Icterus galbula*
	Bullock’s Oriole	*Icterus bullockii*
	Orchard Oriole	*Icterus spurius*
	Yellow-billed Cacique	*Amblycercus holosericeus*
Fringillidae	Domestic Canary	Serinus canaria*
	Euphonia	*Euphonia* sp
	Blue-crowned Chlorophonia	*Chlorophonia occipitalis*
	House Finch	*Haemorhous mexicanus*
	Lesser Goldfinch	*Spinus psaltria*
	American Goldfinch	*Spinus tristis*
	Black-headed Siskin	*Spinus notatus*
	Hooded Grosbeak	*Coccothraustes abeillei*
Passeridae	House Sparrow	Passer domesticus*

^a^An asterisk (*) denotes a species that is non-native to Mexico

Scientific names are fromname the 7th Birds of North and Middle America Checklist

#### Regulation of the activity and the acquisition of permits

The presidents of the unions of *pajareros*–usually referred by union members as the leaders– have the role of mediating between the *pajareros* and the government officers (DGVS) in applying for permits for wild bird capture and bird sale. The leaders inform the union’s members about permit authorizations in periodic meetings in which they help the *pajareros* better understand the administrative and legal contexts that are involved. The *pajareros* have a good understanding of all of the regulation measures applied to their trade, although the interviewees noted that some capturers from remote locations are less aware of such regulations. The interviewees expressed the importance of having official permits for wild bird capture and bird sale, as they know that having permits avoids any legal problems that could create difficulties for their activity. All *pajareros* know the periods of closed seasons that are established by the DGVS. We also identified wild bird conservation practices by communal management, given that the interviewed capturers mentioned “no capture (*no captura*)” of the wild birds during their mating and reproductive seasons, something that they said had existed “forever (*desde siempre*).”

Capture is made during the open seasons; they said that the birds must not be captured during their “heat (*brama*)” period –referring to the mating and reproduction periods– because the birds are “broody (*culecas*)” during that time. In doing this, the *pajareros* ensure that the wild birds are not depleted, thus conserving the birds. Capture generally initiates in July and continues until September and, during this period, the only wild birds that are captured are fledglings, which the *pajareros* call “spotted (*pintos*)” or “new (*nuevos*).” According to the interviewees, if an adult bird of either sex, called “old (*viejo*),” is caught in a trap during these months, it is released, because these birds are reproducing. The parrot use described by Beissinger [61] demonstrates that harvesting young birds with low survival rates causes less of an impact on the bird populations. *Pajareros* recognize females as “the mother (*la nana*)” that is “brooding the eggs (*arrulla los huevitos*)” and males as “the father (*el tata*).” In mid-September, wild birds of all ages are captured, although the *pajareros* insist on protecting the females, because they prefer retaining only the fledglings and males. The fledglings better adapt to captivity and the males are more valued for their song.

#### Capture

The capture of wild birds occurs early in the morning, and the methods used depend on the species of interest and the type of habitat, vegetation, and topography. The sites of capture are communal –*pajareros* call them *postura* or *paraje*, which are both Spanish terms referring to a place in general– and have delimited areas with a given name. Capture areas are used more than once, and the collaboration of all the capturers in the region is needed for covering the different trapping sites across the entire area.

The characteristics of the tools used for capture vary according to the target species and include several types of traps with different names (*tramperas*, *sordas*). Wild bird traps belong to one of two classes according to the mode of capture and local tradition: 1) baits, such as wild fruits, or 2) a live bird called a “*cabresto*” –a term applied to male song birds acting as lure in traps. *Cabrestos* need to be properly fed and are protected from predators during the capture; the wild birds are offered wild fruit as an additional lure. Other studies in Brazil describe several bird capture methods; the most similar to the method used by Mexican *pajareros* is the “alçapão” or “assaprão,” which also uses wild fruit and a live bird as bait [1, 52, 62]. While the use of torches at night and birdlime is common in Brazil (e.g., [53, 62]), we did not find this capture technique in Mexico, and the birds were only used as pets in Mexico and never for blood-sporting (fighting birds) [47, 52].

Once the capturing scene is set, the *pajareros* retreat from the site in order to “hide from the birds (*esconderse de las aves*)” and wait. The capturers, in general, remain attentive and aware of what is occurring in the surroundings in order to interpret what is taking place at the trapping sites. We found similarities to the Yucatan capturer children, who first watch and listen to identify if the birds are present and, if there are predators, the children change place to avoid predation [63]. Trapping can be active, in which the traps close with manual triggering mechanisms, or passive, in which the traps are checked when the *pajareros* hear the noise of the falling trap “lid (*tapa*).”

The respondents who considered themselves to be capturers were male informants (Fig. 9a and d), as was found by Silva Souto and collaborators in Brazil [47]. These authors mention that 85% of the bird trappers were men and, [20] in Bolivia, they interviewed only men trappers. Nevertheless, in Mexico, some women also trap birds in their own yards or at some distance from their homes or community. Women also stated they accompanied their husbands in capturing hikes, but only those at short distances away from their house or community. In fact, the women’s capturing efforts were underrepresented, or their real number was unknown, given that women do not call themselves capturers and the women’s way of capturing birds “is not trapping (*no es trampear*).” Only two women admitted that they were wild bird capturers (Fig. 9b), doing it on their own. Most of the women that had not been on capture hikes said they would like to do it.

Finally, we underline some of the qualities required by *pajareros* for accomplishing the task of capturing wild birds: 1) perseverance for capturing in a constant way, 2) patience for waiting for hours during capture, 3) diligence for waking up early, 4) good physical condition and sufficient agility for walking through forests and hills, 5) physically resistance to fatigue during performance of the activity.

#### Manufacture of nets, traps, and cages

We identified 14 types of wooden frameworks. The type depends on the specific needs of capturing, acclimating, transporting, maintaining, and selling the birds (Fig. 10), the geographic location, and the bird species that one wants to attract or accommodate. The wooden frameworks are manufactured manually, generally using local species of trees and shrubs (Fig. 9c). In the case of trapping the house finch (*Haemorhous mexicanus*), modified fishing nets are also used in a noticeably sophisticated method that involves the design of a “stage” of agave leaves arranged to appear as real agave plants; nets and lures are set in the stage, the latter of which includes stuffed specimens of a male house finch, live *cabrestos* inside a concealed cage, and seeds (Fig. 11). In some communities, capturers made the traditional luring method more technically sophisticated by introducing speakers for reproducing bird songs, which are used together with live *cabrestos*, and even in standard mist nets. It must be stated that many *pajareros* expressed their dislike for the use of mist nets, or their opposition to it. All of the trapped wild birds that are different from the target species are immediately released.

#### Acclimating and keeping the birds

*Pajareros* classify the birds according to their age and degree of adaptation to captivity in two classes: 1) domestic birds that are exotic and are bred in captivity, such as canaries, lovebirds (*Agapornis spp*.), and parakeets; or 2) wild birds born wild. The wild bird class is subdivided into fledglings (*pollos*), which are birds that are too young to fly; and untamed birds (*broncos*), which are recently captured wild birds that are not yet adapted to captivity. The *broncos* are further classified according to age as untamed adults (*broncos adultos*) and untamed young birds with juvenile plumage (*broncos pintos*) [64]; twitters (*gorjeadores*), or birds that are able to emit sounds but are not yet able to sing; and singers (*cantadores*), or birds that are old enough to sing inside their cages, which are further subdivided according to their use either at home or in the field as a lure for capturing wild birds, in which case they are called *cabrestos* (Fig. 11). *Pajareros* differentiate between the maintenance of domestic and wild birds, given that they have different feeding needs. Wild birds require more care and are more delicate than domestic birds.

The amount of time and care required by a bird will depend on its classification (Fig. 12). A study made in the community of Villa Luis Gil Pérez in the state of Tabasco by Parcero Vázquez and Trejo Pérez [65] also reports that the food and care given to Psittacids differs according to the growth stage of the birds. Domestic birds are easier to keep, whereas newly captured wild birds require complicated maintenance measures due to their fragility. The more delicate task of the *pajarero* trade is wild bird acclimation, which requires its mastery by means of learning through experience. Captured wild birds must adapt to a radically different environment and –above all– to different feeding habits. To that end, newly captured wild birds (*broncos*) are subjected to a taming process (*maseado* or *amansado*), which can last from 4 days to a month, during which birds are kept isolated to avoid stressing them due to fear of people (“*para que no se espanten*”). During the taming period, the birds must be regularly monitored and cannot be sold; therefore, the *pajareros* (men or women) must be constantly alert, they must provide regular attention to the birds, and they must train the birds to eat new foodstuffs. Frugivorous birds first need to be fed berries (*frutilla*) from several plant species that they eat in the wild (*frutear*), which, in the first day of captivity, are offered attached to their peduncles or branches to further imitate their natural food. *Pajareros* referred to cases in which *broncos* became depressed from stress, saying that the birds became “spoiled and sad (*se chiquean*, *se ponen tristes*),” which they prevent by covering the cages with a white cloth and adding antibiotics to the drinking water. If a bird that is being acclimated gets wet, it is dried, and if it is dirty, it is cleaned. The first day of captivity is the most important; because of this, the *pajareros* are present the entire day to observe signs of stress in the birds, saying that they see if the birds “fluff up their feathers or become sad, their eyes are not teary and that they are satisfied and lively, and above all, that they eat (*estén esponjados o tristes, que no tengan los ojos llorosos, y que estén contentos y alegres y sobre todo que coman*).” *Pajareros* need to watch the birds handle food appropriately and ensure that they are eating every 15 min. Depressed birds that stop eating –referred to as birds becoming “spoiled (*se chiquean*)”– need special care that consists of separating them from other birds, keeping their cage covered, and providing food directly into their mouths. If a *bronco* refuses to eat properly for 2 days, it is released. Although Alves and collaborators [53] reported that birds suffer high levels of mortality in the first days of captivity in Brazil,; we did not observe significant mortality in the birds managed by the Mexican *pajareros*. For example, in 4-week field trip, we observed one or two recently captured birds that died out of 200 individuals. Foodstuffs provided to birds also vary seasonally; in particular, when the birds molt their feathers (*pelechan*), they are fed dried aquatic insects (*mosco*, a preparation of Prehispanic origin that was formerly also eaten by humans, [66]). In Brazil, the bird keepers also feed insectivore birds some insects [47].

The process of feeding newly captured wild birds consists of initially feeding them a mixture of wild berries (*frutillas*) and a paste made with plantains (*plátano macho*, *Musa balbisiana*), chicken food, and boiled hen egg –a process called *frutearlos* in Spanish, which literally means giving them fruit. In the following days, the proportion of plantain paste is increased as the content of berries is gradually diminished; this process ends when the birds become *plataneros*, meaning that they feed on plantain paste.

However, all birds in captivity constantly need food and water, regardless of how they are classified. It is also essential to know how to prepare bird foodstuffs, given that adequate nutrition is one of the most important factors during early acclimation. Most foodstuffs currently given to birds are the same that have been used by *pajareros* in Mexico for the past 35 years [18]. We did not record the quantities and diversity of food, but, in Brazil, Alves and collaborators [51] provide the proportion of seeds, balanced rations, fruits, vegetables, meat and other items that bird owners use to feed their birds. Some interviewees also mentioned the need for getting up early in the morning to feed the birds, one saying, “the animals eat first (*los animales comen primero*).” Additional nursing measures are ensuring that birds do not injure themselves inside the cage; covering cages on time; keeping birds at an appropriate temperature; preventing predation of birds by cats, rodents or snakes; and curing birds when sick, first by recognizing the symptoms and, afterwards, by giving the birds patent medicines, food supplements, or other remedies (Figs. 13 and 14).

The women involved in bird acclimation explained the laborious and complex tasks that are needed for the maintenance of *cabrestos*. For a bird to become a singing lure (*cabresto*), it must be first sorted among young, newly captured wild birds (*pintos*) and, after about 5 months or a year, the keepers will know which bird will be a *cabresto*; generally, a small fraction of the birds initially chosen as candidates become *cabrestos*. The *cabrestos* “must be taken out to the woods (*hay que sacarlos al monte*)” because it is thought that if this is not done, the bird –although already able to sing– may stop singing. *Cabrestos* must also be provided with special food and their keepers must know how to cure them when ill, for example, when they have a “sore throat (*ronquera*).”

The above description of the acclimation tasks performed by specialists in the trade demonstrates their deep knowledge and extreme care. Acclimation specialists are mostly women, and they are sensitive teachers who are diligent and intuitive (Fig. 15); similar findings have also been reported for keepers of cracids in other communities [6].

#### Sale

Bird sellers offer buyers a recorded number of 96 species of birds (Table 2) in permanent and intermittent markets (*tianguis*, [67]) throughout Mexico, as is common in other Latin American countries (e.g., 41, 53). Carrying cages with a wide variety of birds is a requirement for a good sale. Offering songbirds is a convenient sale-boosting method, given that the songs attract more customers during the workday. Selling trips to other states are frequent, but they may depend on the market demand and weather conditions.

It is convenient that sellers establish a good relationship with their potential buyers, which is expressed by friendly communication. Sellers are good conversationalists and “open conversation (*dan plática*)” by explaining the specific needs of the birds they sell. We identified the bird sellers’ skills of persuasion and for establishing empathy with the buyers. We also observed teamwork capabilities expressed in their common agreement with other buyers for traveling to markets and sharing the transportation costs. In general, the spaces in which birds are sold function as social cohesion places, in which culture and knowledge are exchanged along with the commodities [67].

Based on the Calendar of Songbirds and Ornamental Birds (Calendario Aves Canoras y de Ornato), a listing of the birds used for subsistence in Mexico that is published by SEMARNAT, we estimated that the average monthly income of a bird seller is below the minimum wage (approximately US$135). Because of the low level of income of *pajareros* in Mexico, it has been suggested that prices of birds should be increased to reach to a fair price. Fair prices for birds could be established by means of certifications or sustainability seals issued by SEMARNAT (General Law of Ecological Equilibrium, LGEEPA, Article 77 Bis, Fraction V), which has also been suggested in Indonesia [68, 69], where offering birds with a high quality of song has also been applied for the same purpose [70]. In Mexico and Brazil [47, 52], “cabresto” or living lure birds from the same species, are much valuable than tweeter birds and juvenile birds because of their quality songs. Therefore, promoting the sale of “cabresto” and “cantador” birds would benefit the economy of the *pajareros* families and bird conservation, as *pajareros* would need to capture fewer birds and would devote more time to bird care.

### *Corpus*: knowledge

To succeed in their trade, the *pajareros* need an understanding of several matters, ranging from the biotic and abiotic environment, as shown in Brazil [52], to the socioeconomic, administrative, and legal aspects of the bird trade. Such diverse knowledge is needed because of the varied tasks involved in the activity, including capture, manufacturing of tools, and the acclimation, maintenance, and sale of birds, on top of understanding the administrative and legal processes for obtaining permits (Table 3). Our results (described in the preceding section) indicate that the *pajareros* have deep traditional ecological knowledge (TEK; [71]) derived from a process of learning through doing [72], which occurs during their years of activity in the trade. TEK is not a written knowledge; therefore, its most important intellectual resource is memory and the transmission of what is learned [22]. Our results demonstrate that *pajareros* are specialists in songbirds and ornate birds, which is a knowledge that deserves appreciation by ornithologists, ecologists, veterinarians, and wildlife managers. In the following paragraphs, we describe the knowledge of the *pajareros* in Mexico.

**Table 3:** Synthesis of knowledge on households of *pajareros*

Knowledge	Description of the type of knowledge
Abiotic	Climatology
	Geography: Spatial and topographic knowledge of capturing sites; knowledge of roadmaps and public transportation routes in Mexico; geographic location of markets.
Biotic	Identification of a large number of bird species.
	Biology of captured species: feeding habits, reproduction, etc.
	Ecology of captured wild bird species.
	Predators of captured wild bird species. Identification of herbaceous and woody plants used as inputs. Manipulation of birds: behavior, needed care, diseases. Ethnoveterinary medicine and bird anatomy. Migration of captured wild bird species. Distribution and abundance of captured wild bird species.
Environmental issues	Destruction and loss of habitat, deforestation, land use change, several anthropic activities, and lack of food.
Social	Capability of working in a team. Leadership recognition. Collaborative management of the resources with other capturers.
	Ease of speech, persuasiveness, and empathy with buyers.
Economic	Management of days for capturing. Financial management in buying bird’s foodstuffs and medications. Management of days for selling. Daily management of expenses and budget.
Tool design	Basic carpentry.
Administrative or legal	Environmental laws and norms. Places where capture is banned.
	Species of wild birds that cannot be captured.

Households of *pajareros* know the content of federal environmental laws, such as the General Law of Wildlife (LGVS by its Spanish acronym) and the General Law of Ecological Equilibrium (LGEEPA by its Spanish acronym), and some are experts in the matter. Wild bird capturers are true naturalists who know the biology, habitat, and ecology of their target species in addition to the climate context that limits and modifies these aspects. Because capture is determined by weather conditions, capturers must initially have knowledge about the atmospheric and climate factors. The *pajareros* suspended capture during rainy days, and they must anticipate adverse climate conditions. The capturers choose ideal trapping sites where their target species lives and have a precise knowledge of its habitat requirements. They know the microhabitat preferred by birds for nesting, for example, which are cavities in the margins of rivers and streams. The capturers also know the appropriate trapping places, i.e., where the desired birds are abundant. For example, the trappers know that the slate-colored solitaire is abundant in forested ridges. Additionally, the capturers are concerned with the conservation of wild bird populations and habitats, as their living depends on it.

The manufacturing of wooden frames requires knowledge about the ideal materials, and hence about the local woody plants; it also requires knowledge about basic carpentry, as has been described (e.g., [62, 73]). Knowledge about feeding habits and the behavior of birds, adequate bird handling practices, ethno-veterinary medicine, and economics (for purchasing inputs) are necessary for acclimating and maintaining the captured birds. The bird sellers (Fig. 16) must have knowledge of and experience with the caring measures required by birds, knowing how to identify the bird species that are suitable for being acclimated and sold (Table 2), and be familiar with the national geography.

#### Knowledge of the natural environment of wild birds

The *pajareros* know that wild birds are a natural resource, given that they acknowledge the existing environmental and management issues. For example, they know the factors limiting the biodiversity and abundance of wild birds as a natural resource: a) adverse climate conditions, b) wild bird habitat perturbation, c) availability of the resource, d) overexploitation, and e) other factors of bird biology. In the following paragraphs, we describe each of these factors.

#### Adverse climatic conditions

Both domestic and wild birds are affected by adverse climate conditions. A male interviewee explains that drought leads to a decreased availability of food and, hence, to scarcity of house finches (*Haemorhous mexicanus*). A female capturer explained that hurricanes killed many birds. In locations with cold weather, low temperatures also affect the breeding of domestic birds.

#### Perturbation of bird habitats

A substantial number of interviewees mentioned perturbations from the destruction and loss of wild bird habitats, deforestation, land use changes, and several anthropogenic activities. For example, two *pajareros* stated: “…what is being reduced is the space for birds (…*lo que se está reduciendo es el espacio de las aves*);” and “twenty years ago, there was more forest (*hace 20 años había más sierra*).*”* The more frequently mentioned issue was deforestation, as explained by a *pajarero* “it is logging that is finishing the places; the trees are gone, they were cut down… farmers making their *milpas* …use them for planting (*la tala es la que está terminando con los lugares, ya no son los mismos árboles, los han cortado… los campesinos que hacen sus milpas …los ocupan para sembrar*”. Agriculture, grazing of livestock, and urbanization were also mentioned as part of the problem. Habitat loss also derives from forest fires, road building, mining, and pollution with agrochemicals (e.g., plaguicides, herbicides, and chemical fertilizers, among others). Finally, the *pajareros* mentioned the scarcity of food for wild birds, which was as stated by one as follows: “the forest remains the same, it is due to food, as [the food] sometimes becomes depleted here and they [wild birds] search for it elsewhere (*el bosque está igual, es por la comida porque a veces se acaba ahí y la buscan en otro lado*).”

#### Availability of the resource

The *pajareros* recognize time periods in which the wild birds are scarce, relating such scarcity to three fundamental causes that explain the difficulties in finding the birds. The first cause of the increasing scarcity of wild birds is their decrease in population or their extinction. The interviewed *pajareros* explicitly mentioned four species as examples: the brown-backed solitaire (*jilguero, Myadestes occident*alis), the slate-colored solitaire (*clarín*, *M. unicolor*), the blue-crowned Chlorophonia (*Chlorophonia occipitalis*), and the northern cardinal (*Cardinalis cardinalis*). The interviewees also mentioned wild bird displacement, explaining that wild birds have emigrated either definitely or temporarily, and, consequently, they must be searched for in other places –sometimes far away– or one must wait for them to come back the next year. The displacement of wild birds is thought by *pajareros* to be caused by a lack of food or because the birds “are scared, they are scared away (*se asustan, se espantan*).”

#### Other biological knowledge

Finally, the *pajareros* know the distribution of wild birds and mentioned the species that are absent from their locality or state. In the case of the slate-colored solitaire (*clarín*, *M. unicolor*), they acknowledge their local migration, an event unrecorded in the ornithological literature. They also mentioned the seasonal change in appearance of the wild birds when they molt their feathers (“*pelechan*”), which, as a result, causes them to “look ugly (*se ven feas*)” [64], and customers do not buy such birds. Finally, the *pajareros* stated that the acclimated wild birds do not reproduce in captivity or, if they do, it only occurs in large and costly aviaries.

We conclude that the *pajarero* trade is complex, as it is a traditional trade with specialized tasks, and that the households of the *pajareros* have empirical knowledge that is transmitted within families, almost exclusively from generation to generation (Table 2).

### Kosmos

Birds represent more than a natural resource to *pajareros*; rather, as stated by Toledo [23] for other trades based on natural resources, birds are the center of their existence, the core of their culture, and the source of their identity.

#### Reasons for becoming pajareros

*Pajareros* are motivated to practice their trade by a mixture of interlaced factors, which are grouped as 1) inheritance, 2) tradition, 3) subsistence, 4) lack of alternative opportunities, 5) “only know how,” and 6) enjoyment and satisfaction of being *pajareros*. Each factor group is described in the following paragraphs.

Inheritance of the trade was reiterated by the *pajareros*, as an interviewee expressed by saying: *“*that is what was left to us, as heritage, knowing how to sell birds and have that trade (*eso fue lo que nos dejaron, de herencia, saber vender pajaritos y tener ese oficio*).” Most *pajareros* said that they learned the trade at home from a relative when they were teenagers or children by observation or by participating in the chores of the trade. A capturer said: “[I began in the trade] because of my deceased father. He took us and taught us how we will hang the cages, how we are going to hide so the birds do not see us, and so forth ([yo empecé con el oficio] *por el difunto de mi papá. Él nos llevaba y nos enseñaba cómo vamos a colgar las jaulas, cómo vamos a escondernos para que no nos vayan a ver los pajaritos y así. Ya nos enseñó cómo darles de comer, todo eso*.” The study of Uc Keb and Cervera Montejano [63] in Yucatán reported that children learned how to capture birds from their relatives, above all from their parents. The *pajareros* we interviewed explain that their trade “is from tradition, that is, from far back in time (*es de tradición pues, de muy atrás*).” Other *pajareros* acquired the trade because their spouses came from a family of *pajareros*, and they learned the trade from them. Still others learned the trade from friends or from older *pajareros* that invited them to assist them in the capture or sale of birds, or mutually trained themselves with other apprentices.

Several interviewees said that being a *pajarero* was their only know-how, given that “they devoted all of their life to the birds (*toda la vida la entregaron a los pájaros*);” therefore, practicing another trade was unimaginable to them. As mentioned above, a high proportion of the interviewed *pajareros* did not have access to a formal education, without which they could not work in another activity. An economic need for subsistence was mentioned as another factor in becoming a *pajarero,* as was found in the parrot capturers and sellers in Peru and Bolivia who are in the trade to support their basic living expenses [20]. Among the latter interviewees, some stated that a lack of other jobs made them enter the trade or that they practiced it when not working in other occupations. Some became *pajareros* to receive extra income, with some even stating it was a way of saving, as they held the birds to sell them in times of economic need. However, some said that they “are *pajareros* not by choice, but by need (*no por gusto somos pajareros, si no por necesidad*);” however, many expressed that they entered the trade because they were attracted to it when young. All these motives for being *pajareros* agree with those reported by the studies of Bobo and collaborators [74] in Cameroon and by Wolff and Duarte Silva [75] in Brazil.

#### Satisfaction from the trade

To understand the trade of *pajareros* it is important to consider the satisfaction that they receive from their work. Most of the interviewed *pajareros* acknowledged the advantages of the trade, always considering their individual and collective circumstances. First, the feeling of belonging to the trade motivates *pajareros* to continue in it, as was described by an interviewee: “I tell my sons that, until my death, I will be a *pajarero* (*Yo les digo a mis hijos que hasta la muerte voy a ser pajarero*).”

It is highly important that *pajareros* enjoy practicing their trade due to the multiple reasons that motivate them to continue proudly being *pajareros*. Their accounts identify their sense of belonging to the trade; for example, some said that they liked birds since they were children, and a 72-year-old bird seller said it is the only thing he enjoyed doing. Feeling proud of their trade, all *pajareros* expressed their attraction to the birds, which was reflected in their descriptions of how charmed they are by the birds and how much they enjoy their songs, making their work pleasant and gratifying. *Pajareros* also humanize the birds and describe their satisfaction in knowing the care that the birds need, their mastery of knowledge on what and how to feed the birds, the birds’ required measures of attention, and the birds’ illnesses and remedies. The capturers express their satisfaction because the trade allows them to be in contact with nature: “being here and there, in the hills (*el andar aquí y el andar allá, en los cerros…*),” or, as stated by a capturer: “[I like] mornings in the wilderness, the concert of singing animals, pure air; it is nice, truly nice. It is what I like ([a mí me gustan] *las mañanas en el monte, la cantadera de animales, el aire puro, es bonito, la verdad es bonito. Es lo que me gusta*).” Capturers have fun and relax, as their job is not monotonous and it allows them to get out of their homes; they enjoy capturing birds because they exercise and see it as a sport. Even though the capturers do not always trap birds, they enjoy being outdoors. For these reasons, the *pajareros* have afondness for birds, are pleased to see them, enjoy their colors, like listening to their songs, enjoy caring for them, feeding them, and protecting them, and, in general, they enjoy their daily proximity to the birds. In the study in Brazil [51],] 98% of the interviewed bird keepers expressed a strong attachment to their birds, and as mentioned by Uc Keb and Cervera Montejano [63] in their studies with children, children become attached to and fall in love with the birds that they capture.

*Pajareros* also say that they like caring for wild birds, given that they occasionally discuss sowing the wild plants that the birds use as food in nature. Many also expressed their enjoyment of selling birds because of what it implies, i.e., going away from home, traveling, and visiting new places. The bird sellers said that they know a number of market places and many cities and states; some even bragged about knowing the whole country. Their trade allows them to spend time together with colleagues and relatives and to converse and become acquainted with numerous persons. Satisfying their customers makes the bird sellers happy.

Finally, in economic terms, we determined that the *pajareros* like their work because they are independent. They also state that they earn more money as *pajareros* than in other jobs, such as in factories, and that it is also less arduous than other occupations, such as building.

#### Religious events

Spiritual values are also involved in the trade of *pajareros*, as described in Roldán-Clarà and collaborators ([76] in press), which discusses the cultural and family events organized by the families of *pajareros* and the gatherings in religious pilgrimages and ceremonies in which birds play a central role. During religious events, the birds transform their economic value into symbolic and cultural meanings, becoming of spiritual value. Pilgrimages in which the *pajareros* participate have been taking place in several regions of Mexico since at least 1634 [77, 78] (Fig. 17). Hundreds of *pajareros* carrying their cages decorated with flowers participate in the pilgrimages. One example was the pilgrimage to visit the Basilica of Guadalupe in 2013, which was attended by an estimated 280 families of *pajareros*. The purpose of these pilgrimages is offering the best songbirds to the Virgin of Guadalupe and God –generally mockingbirds (*cenzontle*), slate-colored solitaires (*clarín*), and brown-backed solitaires (*jilguero*)– to sing to the Virgin and be blessed. The meaning of these ceremonies is showing gratitude and asking for the continuation of the trade. A *pajarero* recalls the legend of the miracle of the Virgin of Guadalupe: “the first song that Juan Diego heard was from the birds in the Tepeyac. He listened to the tweets of the birds and said: why, if here there are no flowers or birds? Because of that, the tradition is [offering] birds to the Virgin and we do it in her honor and [the honor] of God; and there our tradition continues… (*lo primero que oyó cantar Juan Diego fueron los pajarillos en el Tepeyac. Él oía los trinos de los pájaros y dijo: ¿por qué si aquí no hay ni flores ni pájaros?, por eso la tradición son las aves para la Virgen y en honor a ella lo hacemos y a Dios; y ahí sigue nuestra tradición…*)”.

## Conclusion

We found that the development of the *pajarero* trade involves the interaction of two main components: 1) traditional ecological knowledge (TEK), which, in this case, consists of being in contact with birds and their habitat; and 2) social structure [79], which is provided by kin and social relationships and by the strong structure of the *pajarero* trade unions, as has also been demonstrated for other trades and places (e.g., [80]).

Rescuing TEK is highly valuable for understanding the biology of species that are not studied by scholars [81]. TEK and scientific knowledge are complementary, together providing a deeper understanding of the biology of species [82]. Such is, for example, also the case in the study conducted in Canada by Gagnon and Berteaux [83], in which they reported that empirical knowledge of the local communities broadened the spatial and temporal scales of the scientific knowledge on the ecology of the Arctic fox (*Vulpes lagopus*). For this reason, it is recommended to document the TEK and social structure involved in the *pajarero* trade in Mexico and Latin America so that governmental institutions and NGOs can make better-informed decisions.

We determined that *pajareros* manage the wild bird resource by establishing consensual internal rules that are enforced by following the instructions given by the leaders through self-controlled behavior and self-vigilance. For example, the members of the community of Roca de Oro, Veracruz, apply social sanctions that consist of shaming the offenders by public exposure. Another measure that was suggested by the *pajareros* is to exert territorial use rights for preventing the use of local resources by foreign capturers. This inner control, self-organization or community-based management is parallel to governmental regulations [84], which has been described in the case of fisheries [85, 86], and which has been proven to be effective for the sustainable management of natural resources as stated by Elinor Ostrom (e.g., [87]), Fikret Berkes (e.g., [82]), and other authors (e.g., [88]). It is therefore important to diffuse the knowledge of *pajareros* and to encourage them to continue with the adequate management that they have practiced until the present in order to avoid capturing birds during their reproduction season or picking nests and chicks. It is also necessary to study and evaluate the traditional management techniques applied to wild bird use, such as the exclusive capture of fledglings and males, and to assess their ecological effectiveness. Additionally, it would be advantageous for the government to recognize the community-based management [85] and to follow each step of bird trade activity.

In summary, our work describes the profile, organization, and traditional practices of households in the bird trade in Mexico and the considerable amount of ethno-ornithological knowledge implied in it. Activities in the bird trade involve capturing, acclimating, maintaining, and selling nearly one hundred species of wild and domestic birds, most of which are native to Mexico. In addition, we describe the cosmovision of the households of *pajareros*, their motivations, and the satisfactions that they find in the bird trade. Overall, we applied an ethnoecological approach to this traditional trade. Birds are a part of the identity of the *pajareros* and a component that gives a meaning to their lives (*kosmos*), which makes them treat the birds in a respectful way (*praxis*) and apply a vast knowledge about the birds’ natural history (*corpus*). We are able to ascertain that the traditional bird trade practiced by the Mexican *pajareros* is a relevant biocultural component that should be preserved, as it is a part of the country’s heritage [26, 89].

## Abbreviations

CONABIO: National Commission for Knowledge and Use of Biodiversity
DGVS: General Direction of Wildlife
LGEEPA: General Law of Ecological Equilibrium
LGVS: General Law of Wildlife
NGOs: Non-governmental organizations
OECD: Organization for Economic Co-operation and Development
PROFEPA: Federal Attorney General for Environment Protection
SEMARNAT: Ministry of the Environment and Natural Resources
TEK: Traditional ecological knowledge
